# Morpho-Physiological Evaluation of *Solanum betaceum* Cav. In Vitro Cloned Plants: A Comparison of Different Micropropagation Methods

**DOI:** 10.3390/plants12091884

**Published:** 2023-05-05

**Authors:** Mariana Correia, Tércia Lopes, Ana Patrícia Puga, Glória Pinto, Jorge Canhoto, Sandra Correia

**Affiliations:** 1 Center for Functional Ecology, TERRA Associate Laboratory, Department of Life Sciences, University of Coimbra, 3000-456 Coimbra, Portugal; 2 Centre for Environmental and Marine Studies (CESAM), Department of Biology, University of Aveiro, 3810-193 Aveiro, Portugal; 3 InnovPlantProtect CoLAB, Estrada de Gil Vaz, 7350-999 Elvas, Portugal

**Keywords:** axillary shoot proliferation, organogenesis, plant physiology, somatic embryogenesis, tamarillo

## Abstract

Tamarillo (*Solanum betaceum* Cav.) is a subtropical solanaceous tree with increasing agronomic interest due to its nutritious edible fruits. Growing demand for tamarillo plants and fruits requires optimization of existing propagation methods and scaled-up systems for large-scale cloning of selected germplasm. Three in vitro protocols have been used to micropropagate tamarillo: (1) axillary shoot proliferation in a semisolid medium, (2) organogenesis, and (3) somatic embryogenesis procedures. Variables such as the age of the established shoot cultures and rooting treatments were also analyzed. The morphological and physiological quality of acclimatized plants derived from all the methodologies were compared, with seed-derived plants used as a control group. Overall, the results show that in vitro-derived plants have a similar development to seed-derived plants. Micropropagation by axillary shoot proliferation was highly efficient, with rooting rates above 80% in most treatments. Organogenesis induction was more effective from lamina explants using MS media with 2.0 mg·L^−1^ 6-benzylaminopurine. Both organogenesis and somatic embryogenesis-derived plants were also morphologically and physiologically equivalent to seed and axillary shoot-derived plants. The specificities of each micropropagation method are discussed.

## 1. Introduction

Tamarillo (*Solanum betaceum* Cav.) is a small solanaceous tree that produces edible and highly nutritious fruits from which processed products might be produced, such as smoothies and jams. Nowadays, consumers are searching for new and nourishing products, and the fact that tamarillo offers a healthy and appealing option shows the potential of this fruit in the market [1,2]. Canada was the top exporter country in 2021, surpassing countries such as Colombia and Ecuador, and New Zealand that used to hold that position [3]. Although still considered an orphan crop, tamarillo has been in considerable demand, particularly in regions with a temperate climate such as Portugal. Nevertheless, a lack of available propagated materials is pointed out by nurseries and producers.

Over the past decades, several plant biotechnology approaches have been used for cloning elite genotypes. One of them is micropropagation, a set of techniques that allows in vitro cloning through plant tissue culture. Micropropagation surpasses some of the problems, such as virus infections [4], presented by the conventional propagation methods, presenting several advantages, such as the ability of large-scale propagation and the production of genetically uniform plants with superior phytosanitary quality [4]. Moreover, micropropagated plants show, in general, more vigor, are more uniform, and present a faster maturation and growth compared with plants of seed origin. There are three techniques of micropropagation distinguishable through the initial material used and the type of response obtained [4]: axillary shoot proliferation, organogenesis, and somatic embryogenesis.

Obtention of tamarillo plants through axillary shoot proliferation and organogenesis has already been described [5,6] and is efficiently used for tamarillo micropropagation [7]. Axillary shoot proliferation presents the advantages of not requiring the induction of new meristems, thus reducing regeneration time and not requiring the formation of *callus,* assuring genetic uniformity. Organogenesis is a technique with a high potential for multiplication given that, in theory, a single explant may originate as many plants as the number of live cells it possesses. Some of its disadvantages are the occurrence of contaminations, oxidation of phenolic compounds, and recalcitrance to regeneration inherent to some species [4]. Finally, somatic embryogenesis (SE), in which, under certain stimuli, somatic cells evolve into embryogenic cells capable of developing somatic embryos and, consequently, plants, has been established [8] and well optimized for tamarillo [9,10,11]. This method allows the cryopreservation of the embryogenic material [12], providing the opportunity of studying the material in the field to test its quality [6]. Additionally, once the embryos have a unicellular origin, this is the most viable technique to regenerate genetically modified plants [13]. Nonetheless, SE presents some limitations associated with a high number of abnormal embryos, low rates of conversion of embryos into plants, and low capacity of attaining embryogenic material from adult explants. If the cultures are maintained for a long period, they may lose their embryogenic competence, which leads to low rates of germination and poor-quality plantlets [14]. SE offers specific advantages such as plant production scaling up in bioreactors and automation [15,16].

Overall, all these in vitro techniques are powerful tools for plant genetic improvement when used in combination with more conventional breeding and propagation techniques [16] and can be used for large-scale cloning of elite tamarillo trees. Nevertheless, the potential gains of in vitro propagation will be negligible whether acclimatized plantlets are not resilient enough to further cope with field conditions [4]. For that reason, it is crucial to optimize in vitro protocols and further evaluate morphological parameters and the physiological performance of the obtained plants after acclimatization. In this context, the main objectives of this work were (i) to conduct a comparative analysis of the different micropropagation methods, including optimization of culture media and different rooting strategies when needed, and (ii) to validate those micropropagation strategies by the morpho-physiological evaluation of the obtained plants. The results obtained may ultimately lead to better-defining crop improvement strategies by contributing informative data on different micropropagation methods and their relevance in identifying high-quality plants for large-scale production or breeding purposes.

## 2. Results

### 2.1. Axillary Shoot Proliferation of NE and LTE Lines, Rooting, and Acclimatization

**Figure 1:**
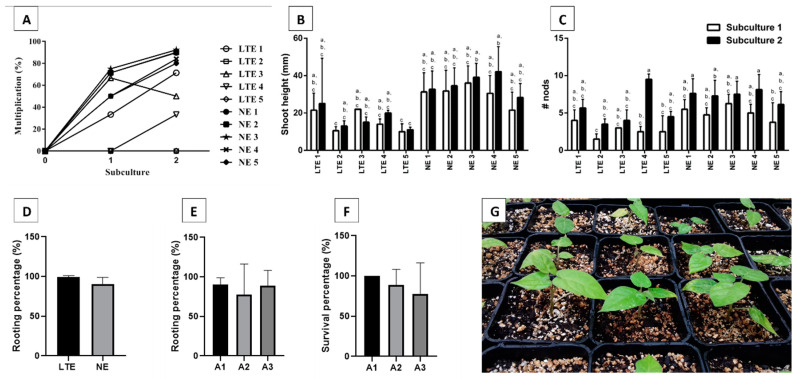
Tamarillo in vitro axillary shoot proliferation and rooting of LTE (long-term established) versus NE (newly established) lines. (**A**) Multiplication percentages; (**B**) shoots height; (**C**) number of nods per shoot; two-way ANOVA was applied, with different letters meaning significant differences according to the Tukey’s test (*p* ≤ 0.05); (**D**) in vitro rooting percentages for LTE and NE; T-test was applied (*p* ≤ 0,05); (**E**) rooting percentages form different rooting procedures (A1–A3); (**F**) survival percentages after 180 days in greenhouse attained; (**G**) plants derived from treatment A1. A1—in vitro; A2—ex vitro; A3—ex vitro 0.2 mg·L^−1^ IBA dipping; one-way ANOVA Tukey’s test was applied, and no significant differences were found according to the Tukey´s test (*p* ≤ 0,05); (**G**) surviving plants after acclimatization.

Tamarillo shoots were micropropagated in a solid medium, and differences between newly (NE) and long-term (LTE) established lines were evaluated. Most NE lines (NE1, NE2, and NE3) attained multiplication percentages higher than 80.0%. In LTE lines, two clones were unable to multiply (LTE2 and LTE5), and the highest multiplication percentage was 71.4% for LTE1 (Figure 1A). NE lines also presented the longest shoots in both subcultures when compared to LTE lines (Figure 1B). A trend towards an increase was observed concerning the number of nodes per shoot from the first to the second subculture in both NE and LTE lines (Figure 1C).

Due to the low multiplication percentages registered for LTE lines, it was not possible to test for different rooting treatments, and only the in vitro rooting procedure was evaluated, allowing us to analyze if rooting capacity was influenced by the age of the cultures. In vitro rooting percentages of LTE lines showed no significant differences (according to Tukey’s test) when compared to NE lines (Figure 1D).

NE line multiplication percentages were analyzed by comparing in vitro (A1) and ex vitro rooting (A2 and A3). In vitro rooting (A1 treatment) showed the highest mean value of rooting percentages, and no significant differences were observed between the different treatments, according to Tukey’s test (Figure 1E). Rooted plants were then placed in the greenhouse, and 180 days later, survival percentages were recorded (Figure 1F,G). Additionally, no significant differences, according to Tukey’s test, were observed between the different treatments, although the in vitro rooting treatment (A1) showed the highest mean value of survival percentages (Figure 1F). Figure 1G shows the plants derived from treatment A1.

### 2.2. Organogenesis Induction Shoots Elongation, Rooting, and Acclimatization

**Figure 2:**
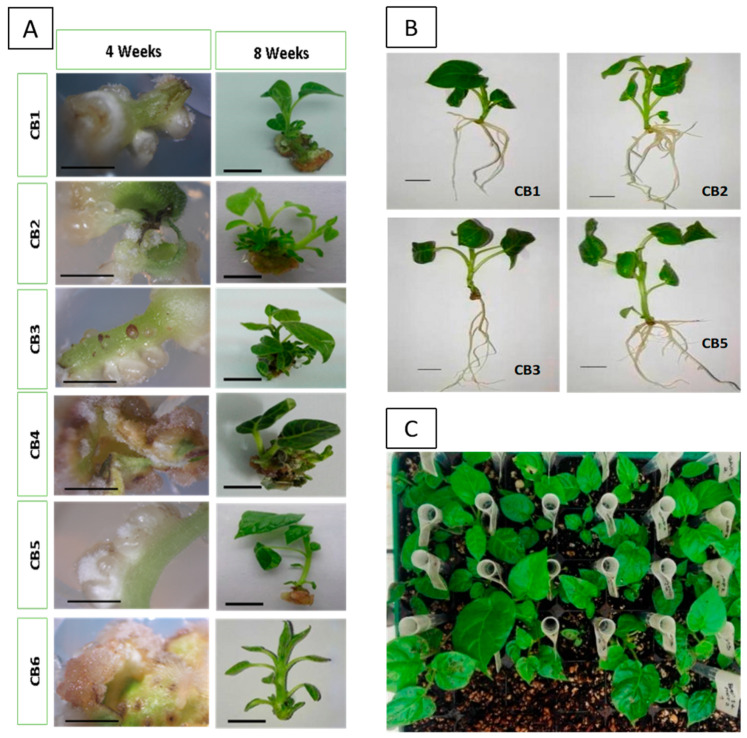
Organogenesis induction (**A**) in red tamarillo lamina and petiole explants in different induction media (CB1–CB6) after four and eight weeks of culture. Induced and elongated shoots were rooted in vitro (**B**) and successfully acclimatized (**C**). Bars = 1 cm.

Tamarillo explants were placed in different induction media, and their induction potential was evaluated over four to five weeks (Figure 2; Table 1).

Overall, lamina explants showed a higher number of shoots and better elongation in comparison with the ones achieved from petiole explants. CB2 medium (2 mg·L^−1^ BA) led to a higher number of shoots and the highest number of successfully acclimatized plants after rooting.

Regarding shoot formation from organogenesis, the results showed that CB2 and CB3 media presented higher shoot formation (Table 1). After four weeks of explant development throughout the induction period (Figure 2), callus induction was present on CB4 (1.0 mg·L^−1^ BA + 2.0 mg·L^−1^ mT) and CB6 (1.0 mg·L^−1^ BA + 0.25 mg·L ^−1^ NAA). The media CB1 (1.0 mg·L^−1^ BA), CB2 (2.0 mg·L^−1^ BA), CB3 (3.0 mg·L^−1^ BA), and CB5 (2.0 mg·L^−1^ BA + 1.0 mg·L^−1^ mT) directly promoted shoot formation (Figure 2A). After eight weeks of culture, well-developed shoots were transferred to the elongation medium.

Besides shoot formation, root formation also occurred on the CB6 (1.0 mg·L^−1^ BA + 0.25 mg·L^−1^ NAA) medium after four weeks of culture. Induction media with the cytokinin BA (CB1, CB2, and CB3) only promoted shoot formation along the explant, and shoot formation was also observed on the media supplemented with both BA and the auxin NAA (Figure 2A).

Table 1 shows the effect of different media on adventitious shoot induction and shoots development. The best results for organogenesis induction were obtained on CB2 and CB3 media, whereas the highest shoot elongation was achieved when CB1 and CB2 were used to stimulate organogenesis in lamina explants. Callus formation was found when explants were cultured in CB2 and CB5 media after four weeks of culture.

The induction of adventitious rooting was also evaluated (Table 2). CB4 and CB6 media did not promote rooting development. All other treatments led to adventitious root formation, and CB1 and CB3 also induced calli formation. CB3 media led to a higher number of roots per shoot. Plantlets derived from induction in CB2 and CB5 media showed a higher average length, and CB5 also led to the longest root length. After acclimatization, all plants derived from successful organogenesis survived.

### 2.3. Assessment of the Embryogenic Capacity of Line ES, Embryo Conversion, and Plantlet Survival

The embryogenic callus previously induced was maintained for four weeks in the development medium, and its embryogenic capacity was evaluated by counting the number of somatic embryos per 100 mg of callus and then their efficient conversion into viable plants. An average number of 22.67 somatic embryos was registered per each mass of 100 mg of *callus*.

**Figure 3:**
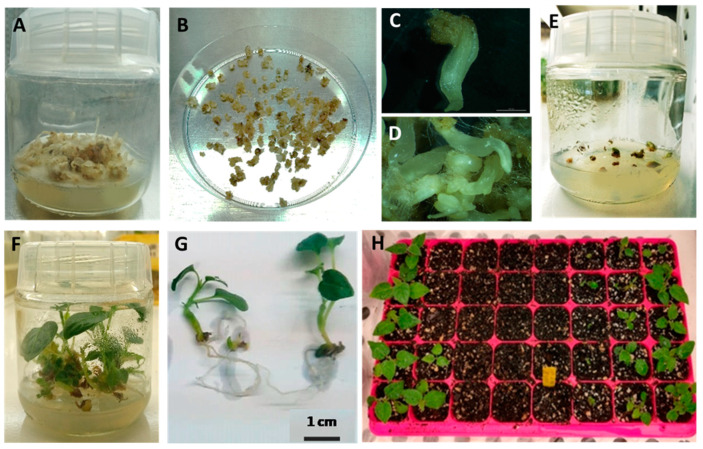
Somatic embryo development and conversion into plantlets. (**A**)—Calli after four weeks in the development medium showing somatic embryos (flask size is 6 cm × 7 cm); (**B**)—Embryos in a Petri dish 100 mm × 20 mm; (**A**)—Morphological aspect of a single somatic embryo after four weeks in the development medium; (**D**)—Cluster of embryos resulting from line ES after four weeks in the development medium; (**E**)—Somatic embryos in the conversion medium; (**F**)—Plantlets resulting from somatic embryos after four weeks in the conversion medium; (**G**)—Somatic embryogenesis-derived plantlets; (**H**)—Plantlets during the acclimatization phase.

Embryo development was not synchronized, and the embryos resulting from the embryogenic line were elongated and did not present any well-defined cotyledons (Figure 3C). Abnormal phenotypes were often observed, and some of them formed tight clusters (Figure 3D).

Overall, the embryo conversion percentages were not high, with 48.15% of embryos resulting from line ES having the capacity to convert into plantlets (Figure 3F,G). Nevertheless, those plantlets showed a survival percentage of 81.25% after acclimatization (Figure 3H).

During the conversion process (Figure 3), it was found that the shoot apical meristem (SAM) developed through a sheath-like structure and that shoots gained a greenish color after being placed under germination conditions. Often the shoots did not develop any foliage and remained in that stage, then turned into a brown color and died without conversion. Frequently, the roots showed a faster development than SAM; therefore, the processes were not simultaneous.

Plantlets did not show a similar development, meaning that some were more evolved than others, and some showed irregular phenotypes, such as twisted stems, stems with bulky appearances, that seemed to be the result of the embryos’ clusters, shoots with reduced growth, and with thick, small leaves when compared with plantlets obtained from seeds.

### 2.4. Physiological and Morphological Parameters from Plant-Derived from All the In Vitro Micropropagation Methods

**Figure 4:**
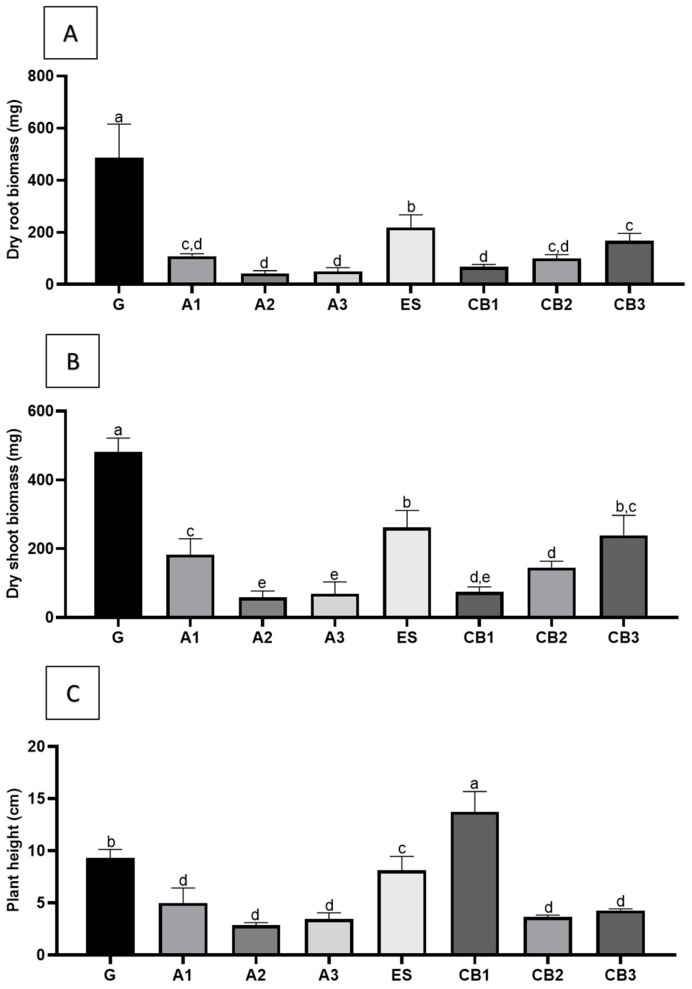
Growth parameters in plants obtained by different propagation methods and seed-derived plants (G), micropropagation-derived plants rooted in vitro (A1), ex vitro (A2), and ex vitro with 0.5 mg·L^−1^ IBA (A3); organogenesis-derived plants in medium supplemented with 1 mg·L^−1^ BA (CB1), 2 mg·L^−1^ BA (CB2), and 3 mg·L^−1^ BA (CB3) and SE-derived plants from the line ES (ES). (**A**) Dry root biomass; (**B**) dry shoot biomass; (**C**) plant height. Means ± SDs, different letters indicate significant differences between treatments, according to Tukey’s test (*p* < 0.05).

As growth parameters, plant height, shoot, and root dry biomass (Figure 4A–C) were registered. The results showed that seed-derived plants (G) were higher and displayed higher values for shoot and root biomasses when compared to the micropropagated plants. The differences were particularly notorious for the biomass parameters. Among the micropropagated plantlets, those obtained through somatic embryogenesis showed significantly higher biomasses than plants derived from either axillary shoot proliferation (A1–A3) or organogenesis (CB1–CB3).

**Figure 5:**
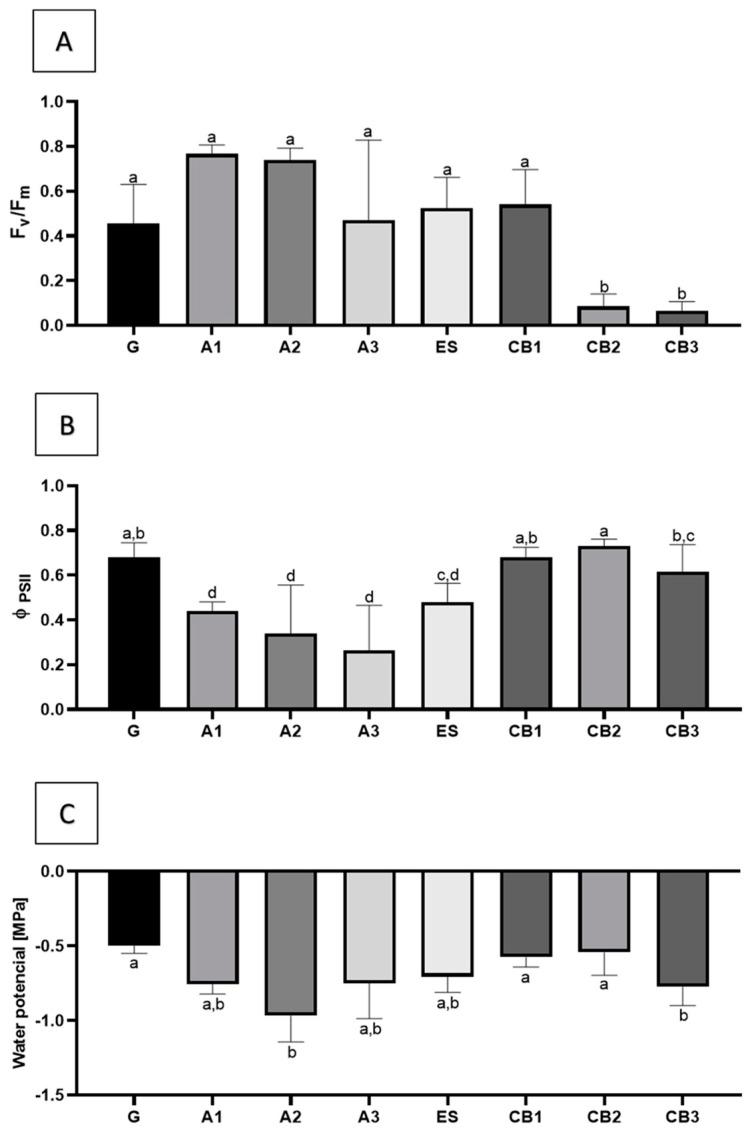
Photochemical parameters and water potential in plants propagated by different propagation methods and in seed-derived plants (G), micropropagation-derived plants rooted in vitro (A1), ex vitro (A2), ex vitro with 0.5 mg·L^−1^ IBA (A3); organogenesis-derived plants in medium supplemented with 1 mg·L^−1^ BA (CB1), 2 mg·L^−1^ BA (CB2), and 3 mg·L^−1^ BA (CB3), SE-derived plants from the line ES (ES). (**A**) Maximum yield of PSII photochemistry—Fv/Fm; (**B**) chlorophyll fluorescence parameters quantum yield of photosystem II—Φ PSII; (**C**) water potential. Means ± SDs, different letters indicate significant differences between treatments, according to Tukey’s test (*p* < 0.05).

Concerning the photosynthetic parameters, Φ PSII (quantum yield of PSII), and the Fv/Fm (maximum yield of PSII photochemistry) ratios, there were statistically significant differences between the different groups (Figure 5A,B). For Fv/Fm, micropropagated plants reached values similar to the control or even higher. However, in some of the plants obtained by organogenesis, significantly lower values were detected, especially for CB2 and CB3 treatments. In the case of Φ PSII, plants developed from axillary shoots showed the lowest values, whereas plants from other origins showed values identical to those obtained in control (seed-derived plants).

The values for water potential (Figure 5C) showed that some organogenesis-derived plants (CB1, CB2) presented values identical to the control, whereas plants from somatic embryos showed lower values, as well as plantlets from axillary shoots.

## 3. Discussion

### 3.1. Axillary Shoot Proliferation and Acclimatization

Very sharp differences were found in the multiplication percentages between LTE and NE lines. Thus, it was found that NE lines had higher multiplication rates than LTE lines, and, in some cases, LTE lines were unable to proliferate, suggesting that in vitro culture time plays a major role in its multiplication capacity.

A decline in multiplication rates following several subcultures has already been observed in several woody species, including cherry (Gisela^®^5 and Gisela^®^6) and plum pear rootstocks [17], shrubs, such as *Cadaba fruticosa* [18] and herbaceous plants, such as *Mentha* spp. [19]. This decrease may be due to genetic and/or epigenetic changes caused by an extended period of exposure to cytokinins, sucrose, or nutrients [18], changes in the endogenous levels of hormones [20], and genotype [19]. This loss of proliferative capacity can be overturned, at least in part, by the maintenance of media culture supplemented with various types and concentrations of cytokinins [18].

Of all cytokinins usually used on micropropagation protocols, BA is one of the most used due to the ease with which it crosses the plasma membrane, catabolism, and promotes the synthesis of endogenous hormones [18]. According to Grigoriado and Maloupa [21], higher BA concentrations lead to smaller shoots with higher node numbers and reduced callus formation. Comparing LTE with NE lines, it can be concluded that LTE has a reduced multiplication capacity due to lower values for shoot formation and lower node formation.

The absence of significant differences between rooting of LTE and NE shoots suggests that rooting, unlike shoot development, is not particularly affected by culture age.

The higher multiplication rates of NE lines allowed us to obtain a greater number of shoots for rooting assays. From the three applied treatments (A1, A2, A3), in vitro rooting gave the best results, although no statistically significant differences were found. Previous work carried out in tamarillo showed that rooting can be achieved in a medium with the auxin IBA [22]. Rooting of hardened plantlets without the addition of exogenous auxin was also obtained by Waweru et al. in *Dovyalis caffra* [23], as ex vitro rooting has been poorly studied in *Solanum*. Our data showed that in vitro leads to better results than ex vitro rooting. However, ex vitro rooting allows simultaneous acclimatization, thus reducing the time of plant regeneration. Ex vitro rooting without auxin was also described for other species such as soybean (*Glycine max*), melon (*Colocynthis citrullus*), marula (*Sclerocarya birrea*), babchi (*Psoralea corylifolia*), and strawberry tree (*Arbutus unedo*) [24,25,26,27,28]. Moreover, performing rooting without the use of auxins helps avoid callus formation and further root differentiation from those calli, which is a condition that, most of the time, leads to a difficult connection between shoots and roots [4].

Survival percentages and rooting percentages expressed higher values for in vitro rooting. However, there were no significant differences. Given that PGR application represents higher costs and in vitro rooting represents a laborious and time-consuming step, ex vitro rooting presents evident advantages to the former treatments.

### 3.2. Organogenesis Induction Optimization and Derived Shoots Elongation, Rooting, and Acclimatization

Shoot regeneration through organogenesis has been described as influenced by different factors such as the growth regulators combination, explant type, and genotype [5,26,27,29]. The action of different growth regulators on explant performance is conditioned by several factors, including species and type of explant used. In the same plant family, a growth regulator can be more effective in one explant type, while in another species, the effect may be the opposite.

In tamarillo, BA was quite effective for de novo shoot induction in red tamarillo explants since whenever BA was used; shoot formation was achieved. Adventitious shoot formation was also induced with meta-topolin (mT) and with NAA. As a rule, segments of the leaf blade showed to be more responsive than petiole segments. The effect of BA on shoot induction has been described in several studies for different species and different explant sources [26,30,31]. It is well known that the auxin: cytokinin ratio plays a major role in organogenesis induction [32]. In red tamarillo, CB4 and CB6 media showed reduced shoot formation capacity and a strong effect on callus formation, which could impair shoot formation, as pointed out for *Scleorocarva birrea* and *Jatropha curcas* [33,34].

Root formation on explants cultured in the CB6 medium may be related to NAA’s high concentration in the medium. The auxin role has been described as essential in meristem organization, promoting the formation of undifferentiated tissue, or differentiated tissues, usually roots [35]. In tissue culture, NAA is mostly used in association with BA for organogenesis induction, and its effects in stem shoot induction are noticeable under lower concentrations. However, in higher concentrations, NAA is mostly associated with callus formation [27], which eventually inhibits shoot and root formation [33,36,37]. In this study, the CB6 medium led to shoot and root formation in the initial stages of induction. However, in the rooting stage of the experiment, there was no adventitious root development, which may be related to NAA’s presence in the media. CB4 promoted callus and shoot formation in the rooting stage but no root development. Shoot formation was observed in CB1, CB2, CB3, and CB5, as well as successful rooting.

Shoots developed in the CB2 medium were rooted better than shoots developed in other media. Several works have shown the potential of mT to more effectively substitute other cytokinis on organogenesis induction [38]. However, in tamarillo, results showed that BA is more effective than mT in the induction stage, a situation which was also recorded for other species such as *Citrus sinensis × Poncirus trifoliata* [39,40]. However, meta-topolin is associated with rooting promotion, and CB5 media, which combined BA and mT, led to interesting results in the rooting stage [41].

### 3.3. Embryogenic Capacity

When the development of embryos from the embryogenic line was assessed, it was found that somatic embryo development occurred, although a great number of somatic embryos showed some abnormality. One of the SE limitations is exactly the low quality of somatic embryos developed from the embryogenic calli, which may lead to difficulties in the stage of conversion into plantlets [42]. These anomalies have already been described in tamarillo, as well as their association with the conversion of embryos into plantlets [43]. The same study also showed that the abnormal phenotypes of the embryos did not impair their conversion into plantlets [11]. This may explain the constraints observed in the stage of conversion for the emblings, showing a differentiation disturbance [42]. The results obtained in this work showed that the ES line mostly differentiates abnormal somatic embryos. In spite of this, conversion was not particularly affected.

As stated before, a two-step SE is a promising biotechnological tool but has limitations, such as the loss of the embryogenic ability of lines when subcultured for a long period of time [44] and the low conversion rates of embryos into plantlets in woody species. Since SE is such a complex process that relies on different kinds of interactions [45], these problems may relate to DNA variations occurring in long-term embryogenic calluses [46] or even embryo anomalies, such as defective accumulation of storage compounds [47].

Overall, there were anomalies in the embryos, and the conversion rate was not very successful. Some of the plantlets obtained from the embryogenic lines died during acclimatization. Even so, the survival rate for the SE plant was still very high, suggesting that even with irregular morphologies in the initial developmental stages, the plantlets may still grow into viable and vigorous plants.

### 3.4. Analysis of Physiological and Morphological Parameters of the Cloned Plants

Some differences in plant height, shoot, and root biomasses were registered between plants obtained from different micropropagation methods when compared with the seedlings-derived plants (G group). Plantlets obtained from seed revealed significantly higher shoot and root biomasses when compared to plants obtained from most other treatments. These lower biomass values for axillary shoot-derived plants may relate to an observed reduction of photosynthetic rates, particularly the quantum yield of photosystem II, compared with seedlings and emblings analyzed. In fact, photochemical performance analysis showed a significant decrease in the effective quantum yield of photosystem II (PSII) for all the axillary shoot proliferation-derived plants, not observable for plants derived from SE or organogenesis. *Lycopersicon esculentum* seed-derived plants and micropropagated plants showed no significant differences in parameters such as PSII, transpiration rate, and stomatal conductance, with the difference that these tomato plants were rooted in vitro [48]. Since the rooting process for plants derived from axillary shoot propagation was induced throughout acclimatization, this may suggest that plants were probably more stressed, which is also reflected in the water potential (Ψ_w_).

Between the remaining groups, the SE-derived plants from the line ES revealed higher values for shoot and root biomass, indicating that the somatic embryos, resulting from the ES line converted successfully into vigorous plants, comparable to plants derived from seeds, as well as the CB2 group, derived from organogenesis. For plant height parameters, CB1 revealed the highest value. Even though there are significant differences, there is a similarity in biomass and plant height between all methodologies and treatments.

In *Coffea arabica,* it was also observed that SE emblings were more vigorous than seedlings, and the observed vigor in the nursery was carried over to field performance as these plants were more precocious than seedlings and yielded coffee beans one year earlier than seedlings [49]. Field performance of *Theobroma cacao* propagated via SE also showed normal phenotypes in field conditions and similar morphological parameters when compared to plants propagated by traditional methods [50]. The Fv/Fm (maximum yield of PSII photochemistry, i.e., the quantum efficiency if all PSII centers were open) results showed that plants derived from organogenesis (CB2, CB3) suffered more with the acclimatization process. The Φ PSII (chlorophyll fluorescence parameters quantum yield of photosystem II), a parameter that measures the proportion of the light absorbed by chlorophyll associated with PSII that is used in photochemistry [51], also showed significant differences between groups, but most of them reached control levels. 

Taken together, these data show that micropropagated plants display physiological parameters similar to those obtained from plants derived from seeds, showing that the in vitro processes do not have any impact on the performance of the regenerated plants. Although SE-derived plants always revealed values lower than seed-derived plants, they maintained consistent results, hinting that once the embryo conversion stage is surpassed, the resulting emblings will survive acclimatization without difficulties. Moreover, micropropagated plants have the advantage of being disease-free and genetically uniform.

## 4. Conclusions

Micropropagation techniques are valuable tools that allow the fixation of particular genotypes and large-scale production. In vitro, axillary shoot proliferation is one of them and the most efficient, but this report showed that successful proliferation is strongly conditioned by the duration of the in vitro culture. Nonetheless, rooting is easily achieved and does not seem to be affected by this factor. The data also showed that ex vitro rooting is clearly more advantageous and cost-effective than in vitro rooting.

Organogenesis induction was more effective in lamina explants and the MS media with 2.0 mg·L^−1^ BA showed better results. Compared to all the tested treatments, this one was the most consistent. The inclusion of the auxin NAA on the organogenesis induction medium promotes callus formation and inhibits shoot growth and further rooting of the induced shoots.

One of the limitations of SE is the development of true-to-type somatic embryos able to germinate and form plantlets able to grow in the field. For tamarillo, the conversion percentages observed for the established embryogenic line evaluated were low, but most of the emblings survived acclimatization. The plantlets that resulted from the somatic embryos revealed some anomalies, such as twisted and bulky stems but recovered during acclimatization, showing that SE-derived plantlets are able to develop into vigorous plants.

Regarding plant performance analysis, the photosynthetic parameters (Φ PSII and Fv/Fm), as well as water potential (Ψ) results, showed significant differences, with the control group having the higher values for Φ PSII and Ψ followed by in vitro propagation by axillary meristems and somatic embryogenesis-derived plants. Significant differences also arose between groups when plant height, shoot, and root biomass were analyzed. Seed-derived plants showed higher values for biomass. However, between methodologies and treatments, all the plants were similar in this parameter. For the plant height parameter, CB1-derived plants revealed the highest value showing that in vitro-derived plants can reach the morphology of seed-derived plants. Overall, in vitro propagation-derived plants’ performance was very similar to seed-derived plants. Considering that each plant micropropagation method has its own advantages, the choice of the micropropagation method will ultimately depend on the specific needs of the plant breeder or researcher. In the future, it would be interesting to follow these plants in experimental fields to see their development and behavior as adult trees.

## 5. Materials and Methods

### 5.1. Plant Material and In Vitro Establishment

Tamarillo shoots were in vitro established from seeds collected from fruits of red tamarillo trees growing at the Botanical Garden of the University of Coimbra. Seeds were surface disinfected with a 7% (*w*/*v*) calcium hypochlorite solution for 15 min. Then, the seeds were rinsed three times with sterile distilled water. For germination, seeds were placed into MS medium [52] supplemented with 3% (*w*/*v*) sucrose, the pH adjusted to 5.7, and agar was used as a solidifying agent at 0.7% (*w*/*v*). This medium was sterilized by autoclaving for 20 min at a temperature of 121 °C. Germination occurred in dark conditions at 25 °C. For axillary shoot proliferation, apical shoots from the plantlets were transferred to a new MS medium supplemented with 0.2 mg·L^−1^ BA (6-benzylaminopurine), and were kept in the growth chamber at 24 ± 1 °C under a 16 h light/8 h dark photoperiod. Established shoot cultures were monthly subcultured to fresh medium on the same conditions.

Tamarillo embryogenic callus was previously induced from leaf explants of red tamarillo shoots multiplied as described above. Somatic embryogenesis induction in leaf segments requires a TP medium that contains the established MS basal medium [52] supplemented with 9% (*w*/*v*) sucrose, 5 mg·L ^−1^ of picloram and Phytagel (Sigma^®^) as a solidifying agent at 0.25% (*w*/*v*). The pH was adjusted to 5.7 before sterilization, which occurred for 20 min at 121 °C in the autoclave. Each leaf (young apical leaves) was cut into four segments, and each segment was stung on the abaxial side and placed in a TP medium. The cultures were incubated in the dark at 25 °C. After 12 weeks of incubation, the embryogenic tissue formed was separated from the non-embryogenic tissue and transferred to the same medium. The embryogenic callus (line ES) was subcultured each 4–5 weeks in a TP medium with the same conditions in which it was induced, following a protocol described in detail by Correia and Canhoto [9].

To obtain enough propagated material for the comparative analysis of the three in vitro propagation methodologies followed in this work, homogenize the experimental design, and reduce the genotype effect, all plant material was obtained from in vitro established seedlings of the same red tamarillo tree, previously selected.

### 5.2. Axillary Shoot Proliferation

#### 5.2.1. Proliferation Rates of Newly Established (NE) vs. Long-Term Established (LTE) Lines

The first objective of this part of the project was to assess the influence of in vitro establishment age in the multiplication capacity using axillary bud proliferation. After red tamarillo seeds germination and shoot establishment, multiplication capacity was evaluated between the newly established (NE) lines (<1 year) and long-term established (LTE) (>5 years). Five nodal segments and apices (≈1 cm) from shoots of both lines were subcultured every five weeks for ten weeks (t0 = culture initiation, t1 = first subculture—5th week, t2 = second subculture—10th week) in an MS medium supplemented with 0.2 mg·L^−1^ BA and 3% (*w*/*v*) sucrose and kept at 24 ± 1 °C under a 16 h light/8 h dark photoperiod. At the end of five (t1) and ten (t2) weeks, shoots attained from each line were evaluated through the measurement of the following morphological parameters: shoot height, number of nodes per shoot, and number of phytomers. Multiplication percentages at the end of the second subculture were determined by dividing the difference of the number of phytomers between t2 and t0 by the initial phytomers number, multiplied by 100.

#### 5.2.2. Rooting and Acclimatization of Micropropagated Shoots

Different rooting procedures were compared for the red tamarillo shoots (≥ 2.0 cm) attained through axillary shoot proliferation: (A1) in vitro rooting in an MS medium supplemented with 3% (*w*/*v*) sucrose in Combiness Microboxx (565 mL) with XXL filters and kept at 24 ± 1 °C under a 16 h light/8 h dark photoperiod; (A2) ex vitro rooting by directly placing the shoots into a moisturized substrate (peat: perlite 2:1); and (A3) ex vitro rooting with 0.5 mg·L^−1^ Indole-3-butyric acid (IBA) quick (<1 s) dipping treatment. Ex vitro treatments were done in trays covered with a grilled cover that allowed to control humidity (>90.0%). Gradually, grills were opened, and trays were uncovered, allowing simultaneous acclimatization. Trays were kept in a climatic chamber at 24 ± 1 °C under a 16 h light/8 h dark photoperiod with 70.0% relative humidity. In vitro, the resulting plants were also acclimatized by transferring the plants to the same trays and climatic chamber. Rooting success was recorded five weeks (t1) after each treatment (n = 3) by analyzing rooting percentage (number of rooted plants/number of initial shoots x 100), longest root length, adventitious roots number, plant height, and plant and roots biomasses (dry weight measured after seven days at 40 °C).

Surviving plants were then transferred to the greenhouse and watered on a two-day basis for two weeks. At the end of this time, morphophysiological parameters were analyzed. Survival percentages after acclimatization of plants coming from the three rooting procedures were determined by dividing the number of surviving plants by the initial number of plants transferred, multiplied by 100.

### 5.3. Organogenesis

#### 5.3.1. Organogenesis Induction

To induce organogenesis, segments of the leaf blade and petiole of red tamarillo shoots leaves were used as explants. The explants were obtained from the plant material micropropagated, as described in Section 5.1. Different induction media (CB1-CB6), with MS basal formulation, supplemented with 3% (*w*/*v*) sucrose and different combinations of the plant growth regulators BA, metatopolin (mt), and 1-naphthaleneacetic acid (NAA) were evaluated (Table 3). They were assessed on multiwell plates (12 inserters) with eight limbus and eight petiole explants per medium. Leaf disks (0.5 cm diameter) were excised from the lamina (main vein removed), and petioles segments (0.5 cm long) were cut from young leaves collected from 6 to 7 cm high shoots after 4–5 weeks on a multiplication medium. The disks were placed with the abaxial side down in the multiwell inserters.

Multiwell plates with the plant material were placed at dark conditions for a 3-week induction period and then moved to a culture chamber at 24 °C, with 15–20 μmol m^−2^ s^−1^ PAR, and a 16 h/8 h photoperiod. After eight weeks of culture, PGRs influence was evaluated, and the following parameters were recorded: callus formation, shoot formation, and the number of shoots produced.

#### 5.3.2. Shoot Elongation, Rooting, and Plant Acclimatization

After eight weeks of induction, responsive explants (47 in total) were transferred to a new medium supplemented with a lower BA concentration (0.2 mg·L^−1^) to promote shoot elongation. After four weeks of culture in this elongation medium, the 47 shoots, with an average of 1 cm high, were isolated and transferred to a rooting medium, similar to the rooting condition A1 described in Section 5.2, for four weeks. The plants obtained were acclimatized and evaluated as described above (Section 5.2).

### 5.4. Somatic Embryogenesis

#### 5.4.1. Somatic Embryo Development from Induced Embryogenic Callus (EC)

Somatic embryo development was achieved by transferring EC masses of the ES proliferating line (three replicas of 100 mg of EC) into somatic embryo development medium consisting of the MS basal medium supplemented with 4% (*w*/*v*) sucrose, with agar at 0.7% (*w*/*v*) as a solidifying agent and pH adjustment at 5.7 before sterilization for 20 min at 121 °C in the autoclave. The calli were maintained in the development medium for 4–5 weeks until the development of somatic embryos was noticeable. The number of somatic embryos obtained per mass of EC was registered.

#### 5.4.2. Somatic Embryo Conversion and Plant Acclimatization

For somatic embryo conversion, the embryos obtained from the EC were then placed in a second auxin-free medium with the same MS-based basal composition but with lower sucrose concentrations (2.5% *w*/*v*), agar at 0.7% (*w*/*v*) as a solidifying agent and pH adjustment at 5.7 before sterilization for 20 min at 121 °C in the autoclave. The somatic embryos were exposed to a 16 h light/8 h dark photoperiod for four weeks, and the number of derived plantlets was registered. Afterward, the conversion rate was calculated by dividing the number of plantlets attained by the number of embryos registered.

The plantlets were then transferred to a mixture of peat and perlite (2:1) in pots and maintained in a climatic growth cabinet chamber under 16 h light/8 h dark photoperiod and with a 24 °C temperature and 70% relative humidity. Acclimatized plants were transferred to a greenhouse. To calculate the survival rate of the tamarillo plants in the acclimatization stage, the final number of plants was divided by the initial number of plantlets transferred to the mixture of peat and perlite. The morphological parameters of the plants obtained were evaluated as described above (Section 5.2).

### 5.5. Seed-Derived Plants Establishment

To obtain the seed-derived plants, tamarillo seeds were sterilized and placed in an MS medium described in Section 5.1. After germination, the seedlings were transferred to a mixture of peat and perlite (2:1) and maintained in a climatic growth cabinet chamber under 16 h light/8 h dark photoperiod and with a 24 °C temperature and 70% relative humidity. After 4–5 weeks, acclimatized plants were transferred to a greenhouse.

## 6. Analysis of Physiological Parameters

**Figure 6:**
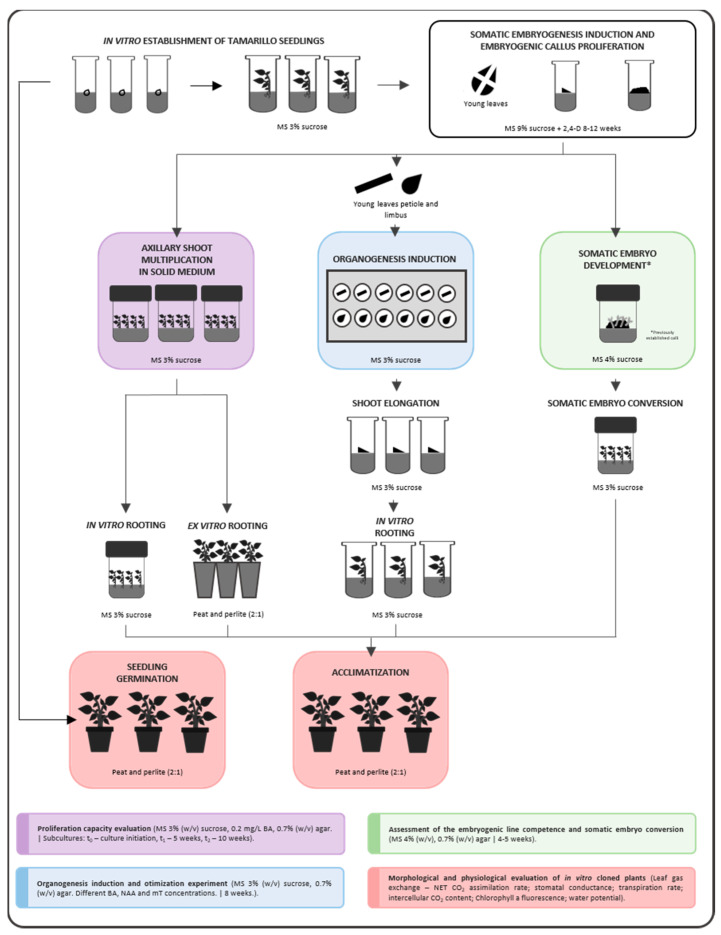
Experimental design followed for the morpho-physiological evaluation of tamarillo plants obtained by different micropropagation techniques. Three propagation methods were tested: axillary shoot multiplication in the solid medium, organogenesis, and somatic embryogenesis. Differences between in vitro and ex vitro rooting and rooting success were assessed for axillary shoot-derived plants. Acclimatization rates were evaluated. Morphological and physiological parameters were measured for plants derived from the three micropropagation methods and for seed-derived plants.

After approximately nine weeks in greenhouse conditions, five plants per each group/treatment were selected to be analyzed, as shown in Figure 6. The eight groups/treatments were: seed-derived plants (G); axillary shoot-derived plants rooted in vitro (A1), ex vitro (A2), and ex vitro with 0.5 mg·L^−1^ IBA (A3); organogenesis-derived plants induced in medium supplemented with 1 mg·L^−1^ BA (CB1), 2 mg·L^−1^ BA (CB2) and 3 mg·L^−1^ BA (CB3); and SE-derived plants (ES).

Chlorophyll fluorescence was determined in situ on the same leaves as used for the gas-exchange measurements with a portable fluorometer (Mini-PAM; Walz, Effeltrich, Germany). Light-adapted components of chlorophyll fluorescence were measured as described in Jesus et al. [53]: steady-state fluorescence (F), maximal fluorescence (F’m), variable fluorescence (F’v, equivalent to F’m-F), and quantum yield of PSII photochemistry [Φ PSII, equivalent to (F’m-F)/F’m)]. Leaves were then dark adapted for at least 20 min to obtain F0 (minimum fluorescence), Fm (maximum fluorescence), Fv (variable fluorescence, equivalent to Fm-F0), and Fv/Fm (maximum quantum yield of PSII photochemistry).

Water potential (Ψ_w_) was determined with a Scholander-type pressure chamber (PMS instrument Co., Albany OR, USA). The height of the plants was also recorded by measuring the plant shoots with the help of a digital caliper. Dry biomass was further assessed after separating the shoot part and root part of the plants and placing them for a period of eight days in a heat chamber at 40 °C. All the experimental procedures described in the material and methods are represented in Figure 6. 

## 7. Statistical Analysis

Proliferation capacity, rooting, and acclimatization evaluation data were analyzed by two-way ANOVA, followed by Tukey’s multiple comparison tests to identify significant differences between means (*p* < 0.05).

The data resulting from the physiological evaluation of the plants were analyzed by a one-way ANOVA comparing the means of each group, followed by Tukey’s multiple comparison tests (*p* < 0.05). All the statistical analyses were performed with GraphPad Prism for Windows v. 6.01.

## Figures and Tables

**Table 1:** Effect of different culture media on organogenesis induction on explants of red tamarillo lamina (L) and petioles (P). Induction parameters were registered after eight weeks of culture. Except for the average number of induced shoots per explant, the presented values correspond to the average value ± SD (n > 8).

Treatment	Average Number of Roots per Shoot	Longest Root Length (cm)	Shoot Average Length after Rooting Stage (cm)
CB1	2.83 ± 1.1	5.33 ± 0.5	1.75 ± 0.7
CB2	2.87 ± 1.7	5.63 ± 1.7	2.97 ± 1.3
CB3	3.13 ± 1.8	5.88 ± 1.3	2.75 ± 1.9
CB4	N/A	N/A	N/A
CB5	2.63 ± 1.3	7.31 ± 2.5	3.00 ± 1.0
CB6	N/A	N/A	N/A

**Table 2:** Rooting phase shoot parameters of the induced shoots lamina (L) and petioles (P) from the different organogenesis induction media after four weeks of rooting media. Except for the average number of induced shoots per explant, the presented values correspond to the average value ± SD (n > 8).

Treatment	Explant	Average Number of Shoots per Explant	Average Shoot Elongation (cm)
CB1	L	0.5	1.25 ± 0.1
	P	0.25	0.87 ± 0.3
CB2	L	1.375	1.58 ± 0.8
	P	0.5	0.62 ± 0.4
CB3	L	0.875	0.87 ± 0.5
	P	0.5	0.67 ± 0.6
CB4	L	0.125	0.98 ± 0.2
	P	0.25	0.89 ± 0.6
CB5	L	0.625	1.00 ± 0.1
	P	0.375	0.86 ± 0.6
CB6	L	0.25	1.35 ± 0.6
	P	0.25	1.10 ± 0.3

**Table 3:** Media composition used for organogenesis induction.

Medium	Composition
CB1	MS + 1.0 mg·L^−1^ BA
CB2	MS + 2.0 mg·L^−1^ BA
CB3	MS + 3.0 mg·L^−1^ BA
CB4	MS + 1.0 mg·L ^−1^ BA + 2.0 mg·L^−1^ mT
CB5	MS + 2.0 mg·L ^−1^ BA + 1.0 mg·L^−1^ mT
CB6	MS + 1.0 mg·L^−1^ BA + 0.25 mg·L^−1^ NAA

## Data Availability

Not applicable.
